# Analysis of the Damage Mechanism around the Crack Tip for Two Rubber-Toughened PLA-Based Blends

**DOI:** 10.3390/polym13224053

**Published:** 2021-11-22

**Authors:** Vito Gigante, Luca Bosi, Paola Parlanti, Mauro Gemmi, Laura Aliotta, Andrea Lazzeri

**Affiliations:** 1 Department of Civil and Industrial Engineering, University of Pisa, Via Diotisalvi, 2, 56122 Pisa, Italy; vito.gigante@dici.unipi.it (V.G.); l.bosi1@studenti.unipi.it (L.B.); 2 Interuniversity National Consortium of Materials Science and Technology (INSTM), Via Giusti 9, 50121 Florence, Italy; 3 Istituto Italiano di Tecnologia, Center for Materials Interfaces, Electron Crystallography, Viale Rinaldo Piaggio 34, 56025 Pontedera, Italy; Paola.Parlanti@iit.it (P.P.); Mauro.Gemmi@iit.it (M.G.)

**Keywords:** poly(lactic) acid (PLA), rubber toughening, microscopy, damage mechanisms, dilatational bands

## Abstract

The toughening mechanisms of poly(lactic acid; PLA) blended with two different elastomers, namely poly (butylene adipate-co-terephtalate; PBAT) and polyolefin elastomers with grafted glycidyl methacrylate (POE-g-GMA), at 10 and 20 wt.%, were investigated. Tensile and Charpy impact tests showed a general improvement in the performance of the PLA. The morphology of the dispersed phases showed that PBAT is in the form of spheres while POE-g-GMA has a dual sphere/fibre morphology. To correlate the micromechanical deformation mechanism with the macroscopical mechanical behaviour, the analysis of the subcritical crack tip damaged zone of double-notched specimens subjected to a four-point bending test (according to the single-edge double-notch four-point bend (SEDN-4PB) technique) was carried out using several microscopic techniques (SEM, polarized TOM and TEM). The damage was mainly generated by shear yielding deformation although voids associated with dilatational bands were observed.

## 1. Introduction

“Rubber toughening” is a process in which rubber micro or nanoparticles are interspersed in a brittle polymer in order to promote energy absorption through the initiation of local yielding [1]. It is well known that the aim of the polymer modification is to develop a blend or composite with higher toughness and increased elongation at break, while retaining desirable properties such as stiffness and strength. This achievement is not trivial because it is necessary to promote energy absorbing plastic deformation through structural modifications [2]. In binary blends constituted by a brittle matrix with rubber domains dispersed in it, the combination of the rubber phase morphology and the polymer chain structure determine the fracture behaviour of the final blend [3,4,5]. Moreover, the effects of disperse rubber particles are complex and differ among amorphous and semi-crystalline polymer systems [6]. 

Within the framework of toughening mechanisms of rubber modified polymers, the studies and observations of micromechanical processes are crucial [7]. Different mechanisms occurs during the deformation of rubber-toughened polymers and it is important to state that micromechanical mechanisms are competitive processes, in which the prevailing one is determined by the inherent properties of the matrix polymer and by the local stress distribution [8]. The dominant micromechanical deformation mechanism is influenced by the molecular rearrangement, composition of the matrix material, test temperature, strain rate, and rubber particles’ shape and size [9]. In addition, it is important to underline that some rubber-toughening theories for multiphase systems are valid just for few systems and, in some cases, they do not explain all the experimental evidence. For example, the multiple matrix crazing theory [10] is focused on the toughening effects of crazing that starts at the equator of rubber particles, but multiple crazing has been actually observed in few polymeric systems, namely styrenics [11]. Even the shear yielding theory [12], which assumes that rubber particles can act as stress concentrators producing shear bands, cannot alone explain how very small particles cannot effectively toughen a polymer blend since the stress concentration is considered the same for small and large particles [13]. Indeed, it is complicated to understand the complex interactions between the different micromechanical processes considering that both energy absorption and energy dissipation processes are important to toughen the materials [14]. 

**Figure 1:**
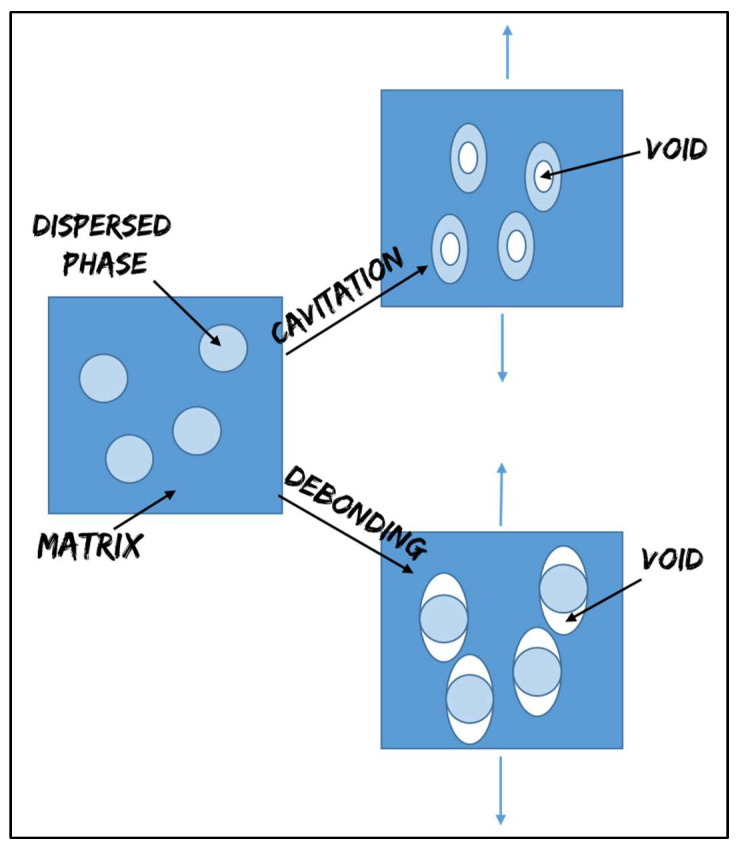
Cavitation and debonding mechanisms.

Therefore, as mentioned above, the cooperation of several micromechanical deformation processes (occurring sequentially or even simultaneously) may effectively toughen the material. In this context, on the basis of energy balances and experimental confirmations, Lazzeri and Bucknall [15,16] developed the idea that the cavitation of rubber particles is the determining step in the rubber toughening mechanism. Cavitation, in fact, arises at the crack tip, followed by void growth. If the voids are generated at the interface between the matrix and rubber particles [17], the mechanism is called debonding (Figure 1). Usually, high adhesion values contribute to the internal cavitation of the rubber particles, while low values contribute to the debonding mechanism. Frequently, it is possible to have a combination of both mechanisms if suitable interfacial adhesion values are achieved [18].

Additionally, dilatational shear yielding and/or crazing in the matrix can occur. The following distortion of the matrix is not homogeneous and becomes highly localized due to the formation of bands of voids: the so-called “dilatational shear bands”. In these bands, the voids are not interconnected but rather the bands are planar arrays of cavitated particles where the matrix between voids is subject to high shear strains and reduced constraints on the plastic flow [19]. This idea was found to be effective, demonstrating the cooperation of different micromechanical deformation mechanisms, and it has laid the basis for the development of the rigid filler toughening theory of Argon and Cohen [20,21].

To inspect and investigate toughening mechanisms, and to understand the correct micromechanical pathway, is necessary the use both electron and optical microscopy [22]. In fact, by using a range of electron microscopy techniques associated with tailored mechanical characterizations, it is possible to reveal and deeply understand the mechanisms of energy absorption for multiphase polymeric systems. Scanning electron microscopy (SEM) is used to inspect fracture surfaces, which are regions of the highest stress and deformation. Transmission electron microscopy (TEM) is used to search for damage that occurs below the surface [23], while transmission optical microscopy (TOM) is often used with crossed polars to detect regions of permanent orientation, i.e., plasticity [24]. In fact, the drawn material of the process zone can be regarded as a second phase since it differs from the original one by its physical properties and is separated by a distinct boundary. A high degree of molecular orientation occurs within the deformation zone and is clearly visible through birefringence. Therefore, where there is birefringence, it is possible to associate a plastically deformed zone, as reported in literature [25,26,27,28].

An interesting starting point to study toughening mechanisms is to observe the damage zone around the surviving crack tip, produced by a mechanical test, called the single-edge double-notch four-point bend (SEDN-4PB) technique [29]. In this method, one crack will reach its critical stress intensity factor and propagate, while the second one does not propagate. This method, therefore, produces a sub-critical propagated crack that can be analyzed by coupling different microscopy techniques [30,31]. 

These studies were firstly applied on rubber-toughened epoxy resins [32,33,34] and, more recently, the SEDN-4PB technique associated to microscopy has also been used to investigate the toughening processes of modified thermoplastics [35,36,37]. Due to their appealing processing advantages, impact-modified thermoplastics, indeed, represent one of the fastest growing types of products in materials industries [38].

In this field, a new trend in polymer research is the application of the rubber-toughening process to biopolymer matrices such as poly(lactic acid) PLA a promising bio-based, biodegradable, and easily processable material. PLA has a high tensile resistance and stiffness but shows some drawbacks to be overcome, such as its high brittleness and low impact resistance [39,40,41]. For these reasons, rubber-toughening processes (with bio-based or biodegradable rubbers) were studied to improve PLA fracture resistance and/or ductility while preserving the eco-compatible nature of the final multiphase material [42,43,44]. In literature, the addition to PLA in different amounts of elastomer typologies, for numerous applications, has been extensively studied from a rheological, processing, thermal, and mechanical point of view, with positive and encouraging results [45,46,47]. 

Nevertheless, to the best of our knowledge, no studies have been carried out on the investigation of rubber-toughened PLA systems by observing (via SEM, TEM, and TOM) the damage zone around the tip of the non-propagated crack produced by SEND-4PB. For this reason, in this work, two different elastomers (PBAT and POE-g-GMA) already known in literature for their capability to toughen PLA [48,49] were investigated, correlating the macroscopically mechanical results to the micromechanical deformation mechanisms observed. 

## 2. Materials and Methods

### 2.1. Materials

**Figure 2:**
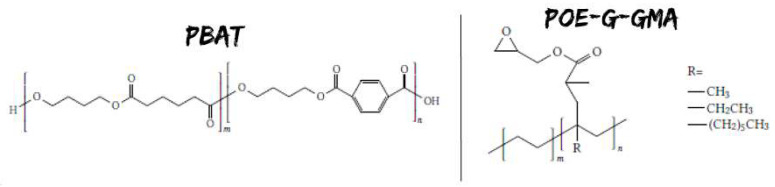
Chemical structure of the monomeric units of PBAT and POE-g-GMA.

The material used in this study were:

- Poly (lactic acid; PLA) Ingeo™ Biopolymer 20003D (thermoforming and extrusion grade) purchased from NatureWorks (Melt Flow Index (MFI): 6 g/10 min (210 °C, 2.16 kg); density: 1.24 g cm^3^; nominal average molar mass: 200,000 g/mol);
- Poly (butylene adipate-co-terephtalate; PBAT) (Figure 2) C1200, trade name Ecoflex^®^, purchased from BASF. It is a fully biodegradable aliphatic-aromatic copolyester based on the monomers 1,4-butanediol, adipic acid, and terephthalic acid (MFI: 2.7–5 g/10 min (190 °C, 2.16 kg); density: 1.26 g/cm^3^, nominal average molar mass: 126,000 g/mol);
- Polyolefin elastomers with grafted glycidyl methacrylate (POE-g-GMA) (Figure 2), trade name SOG-02, purchased from Fine-blend Compatibilizer Jiangsu Co., Ltd. (Nanjing, China) (MFI: 2–5 g/10 min (190 °C, 2.16 kg); density: 0.88 g/cm^3^; nominal average molar mass of 220,000 g/mol and grafted ratio of 0.8–1.2 wt.%);
- Epoxy resin, Elan-tech^®^ EC147/W147, with a low viscosity two-component epoxy system, purchased from Elantas Italia (resin density: 1.13 g/cm^3^; curing agent density: 1 g/cm^3^). This resin was used to embed the four-point bending (4PBD) fractured specimens to “freeze” the cracks for the subsequent sectioning according to the procedure explained in Section 2.2.3.

### 2.2. Methods

#### 2.2.1. Blends and Specimen Preparation

Binary blends with two different amounts of PBAT and POE-g-GMA (Table 1) were extruded in a co-rotating semi-industrial COMAC EBC 25HT (Comac, Cerro Maggiore, Italy) twin screw extruder (L/D = 44). Before the extrusion, all solid materials were dried in a ventilated oven for at least 24 h. PLA was introduced in the main feeder while PBAT and POE-g-GMA were fed separately from a specific feeder which allows for a constant concentration in the melt during the extrusion with a fixed weight percentage to add. 

During the extrusion, the temperatures in the zones from 1 to 11 were 150/175/180/180/185/185/185/185/175/165/165 °C, with the die zone at 165 °C. The screw rate was kept at 270 rpm. The extruded strands derived from the dies were cooled in a water bath at room temperature and reduced in pellets by an automatic knife cutter. All pellets were then dried in a Piovan DP 604-615 (Piovan S.p.A, Verona, Italy) dryer at 60 °C.

After the extrusion, the pellets were injection-molded using a Megatech H10/18 (TECNICA DUEBI s.r.l., Fabriano, Italy) injection-molding machine to obtain two types of specimens: dog-bone samples for tensile tests according to ISO 527-1A (width: 10 mm; thickness: 4 mm; length: 80 mm) and parallelepiped specimens for both the Charpy impact test according to ISO 179 (width: 10 mm; thickness: 4 mm; length 80 mm) and for the analysis of the damage mechanics around the crack tip in the four-point bending test (SEDN-4PB). The processing parameters adopted during the injection molding are reported in Table 2; the conditions were kept the same for all blends to avoid discrepancies ascribed to the different conditions adopted (especially mold temperature, injection holding time, and cooling time). 

#### 2.2.2. Mechanical and Morphological Characterization 

Tensile tests were carried out on ISO 527-1A dog-bone specimens using an MTS Criterion model 43 universal testing machine (MTS Systems Corporation, Eden Prairie, MN, USA) at a crosshead speed of 10 mm/min, equipped with a 10 kN load cell and interfaced with a computer running MTS Elite Software. Tests were carried out after two days after the injection-molding process and, during this time, the specimens were stored in a dry keeper at controlled atmosphere (room temperature and 50% of relative humidity). At least five specimens were tested for each blend composition. 

Charpy impact tests were performed on notched samples. The V-notch in the center of the specimens was made by a manual V-notch cutter (45° V-notch; depth: 2 mm). For the impact tests, an Instron CEAST 9050 machine (INSTRON, Canton, MA, USA) was used. Five samples of each blend composition were tested at room temperature; in this case, the tests were carried out after two days after their injection molding.

Blend morphological characterization was performed on cryo-fractured Charpy samples by FEI Quanta 450 FEG scanning electron microscopy (SEM; Thermofisher Scientific, Waltham, MA, USA). To avoid charge build-up, the samples were sputtered beforehand (with an LEICA EM ACE 600 High Vacuum Sputter Coater, Wetzlar, Germany) with a thin surface layer of platinum. 

#### 2.2.3. Single-Edge Double-Notch Four-Point Bend (SEDN-4PB) Technique 

**Figure 3:**
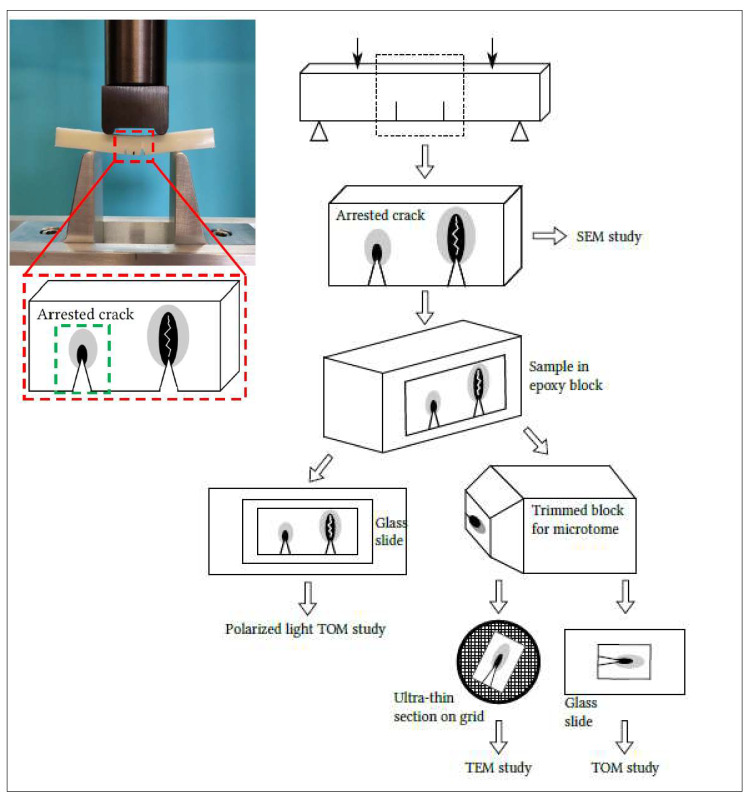
Schematization of SEDN-4PB configuration and of the sampling process for the different microscopy techniques.

The SEDN-4PB technique is known to be very effective in probing the toughening mechanisms of rubber-toughened systems under a triaxial stress state. The technique (shown in Figure 3) consists of bending a specimen that has two almost identical cracks on one side. Due to the intensification of stresses on the crack tips, a plastic zone forms independently on each crack tip at the time of loading (the distance between the cracks is greater than the size of the plastic zone). As the cracks are not exactly identical, one of them becomes critical and propagates in an unstable manner, thus unloading the other crack, which immediately becomes stationary with a subcritical damaged zone. The SEDN-4PB tests were always conducted with the before-mentioned MTS universal testing machine at a crosshead speed of 1 mm/min. Before the achievement of the sharp notches (3 mm depth) with a manual cutter machine, the specimens were first cooled in liquid nitrogen for a few minutes to avoid any eventual heat, generated by the movement of the blade, inducing a plastic deformation in the notches proximities. All the optical investigations carried out on SEDN-4PB tests are summarized in Figure 3.

A preliminary investigation after the bending test was carried out on the surface area of the arrested cracks of SEDN-4PB samples by a FEI Quanta 450 FEG (Thermo Fisher Scientific, Waltham, MA, USA) scanning electron microscope (SEM). The samples were coated, also in this case, with a thin layer of platinum prior to the microscopy to avoid charge accumulation.

For investigating the subcritical damaged zone in the plane strain condition, it is necessary to observe the inner part of the samples through polarized light transmission optical microscopy (TOM) and transmission electron microscopy (TEM). The specimens, after the SEDN-4PB tests, were left to settle down for one day and then were embedded in epoxy resin as well as cured at room temperature for two days in order to “freeze” the cracks movements. 

Polarized light TOM allows for the detection of the areas of the specimen that have undergone plastic deformation. Thin sections were obtained using the petrographic polishing technique [32]. In particular, the sections for polarized light TOM were obtained from samples cut into parallelepipeds 3.6 cm long and less than 2 cm wide, and then, by petrographic polishing, were brought to a thickness of about 50 µm and glued onto sample glass slides. The sections were imaged with a ZEISS Axioplan microscope equipped with a Canon EOS digital camera. 

Samples for the TOM and TEM analysis were carefully trimmed down to 6 mm × 6 mm, forming a block with the arrested crack tip. The block face with the arrested crack tip was further reduced in size to approximately 2 mm × 2 mm for ultramicrotome sectioning. Sections for TOM (500 nm thick) and TEM (120 nm thick) were cut using an ultramicrotome (UC7, Leica Microsystem, Vienna, Austria) equipped with a 45° diamond knife (DiATOME, Nidau, Switzerland) and collected on both glass sides and G300Cu TEM grids (EMS), respectively. TOM imaging was performed using an optical microscope (DM750, Leica Microsystem, Vienna, Austria) equipped with an ICC50HD (Leica Microsystem, Vienna, Austria) digital camera. The TEM analysis was carried out with a ZEISS Libra 120 Plus transmission electron microscope operating at an accelerating voltage of 120 keV, equipped with an in-column omega filter to perform energy-filtered imaging and with a bottom mounted 12-bit 2 k × 2 k CCD camera (TRS). 

## 3. Results and Discussion 

### 3.1. Mechanichal and Morphological Results

**Figure 4:**
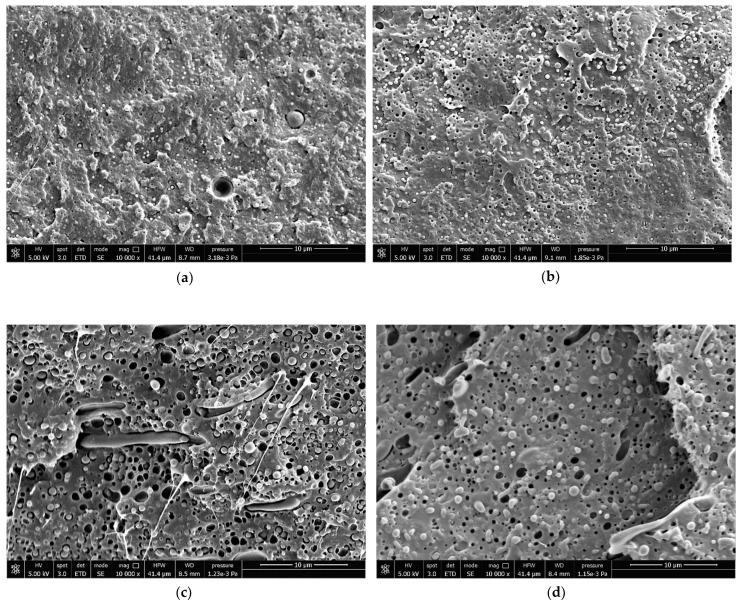
SEM micrographs of: (**a**) P10, (**b**) P20, (**c**) S10, and (**d**) S20.

The blends’ morphology of the cryo-fractured injection-molded specimens is reported in Figure 4.

PLA/PBAT blends (P10 and P20; Figure 4a,b) are characterized by PBAT particles, with particle sizes of less than 1 µm, according to a previous work in which, for the same system, a particle distribution was evaluated [42]. They were sufficiently uniformly dispersed in the PLA matrix. No co-continuity between the phases could be recognized, thus the P blends can be classified as two-phase systems in which PBAT is dispersed in the PLA matrix. With increasing PBAT content, the spherical droplets increase in size according to literature [50,51].

PBAT particles looked sharply defined on the cryogenic fracture surface. Voids were observed at the interface between PBAT and PLA, an indication that debonding had taken place and can be correlated with the fracture advancement through the matrix interface. Considering that the samples were cold fractured, the excessive debonding phenomenon can be associated with the presence of dilatational stresses induced by the thermal expansion coefficient difference between PLA and PBAT, as described by Teamsinsungvon et al. and Jiang et al. [52,53].

The POE-g-GMA/PLA blends (S10 and S20; Figure 4c,d) showed a different morphology. POE-g-GMA was regularly dispersed with a mixed morphology: sphere-like and fibre-like, as observed by Wang et al. [54] and Feng et al. [55]. From a qualitative point of view, the spherical particles had a size slightly greater than PBAT particles. It is possible to observe the voids left by the POE-g-GMA domains having the form of more or less coarse fibres. There were no clear gaps between the dispersed phase and the PLA matrix, suggesting a better interaction between PLA and POE-g-GMA. However, voids were observed at the interface, suggesting that debonding in the minor part with respect to P blends had occurred.

To correlate the morphologies to the macroscopic mechanical behaviour, quasi-static tensile tests and Charpy impact tests were performed (Table 3) and compared to literature data of pure PLA 2003D extruded, molded, and tested in the same lab [56].

From the experimental data for both blends, as could be expected, a decrease of the Young’s Modulus and of the stress at break were detected. Additionally, an increase in ductility (assessed through the analysis of the elongation at break) and Charpy toughness were assessed. The most evident fact was that the two blends showed different behaviours. P blends, as the PBAT content increased, guaranteed an improvement of Charpy impact resistance of up to 50% more compared to pure PLA. Noteworthy for P blends, the remarkable elongation at break increment of up to 270% must be highlighted. This behaviour can be ascribed to the elongation of the voids at the matrix–spherical particle interface. 

Differently, for S-blend the toughness improvement was related tothe impact tests. In fact, the Charpy impact resistance was improved by more than 300% with respect to pure PLA. These results suggest that the poor impact strength of PLA could be significantly improved by blending with POE-g-GMA. In contrast, the elongation at break improvement was not so pronounced as with the P blends. This peculiar mechanical behaviour will be explained in Section 3.2 due to the SEDN-4PB investigation in which PBAT revealed to guarantee a toughening of the PLA subjected to a slow uniaxial test, while the POE-g-GMA had a rubber-toughening effect mainly on the impact test. 

### 3.2. SEDN-4PB Results

#### 3.2.1. SEM Results

**Figure 5:**
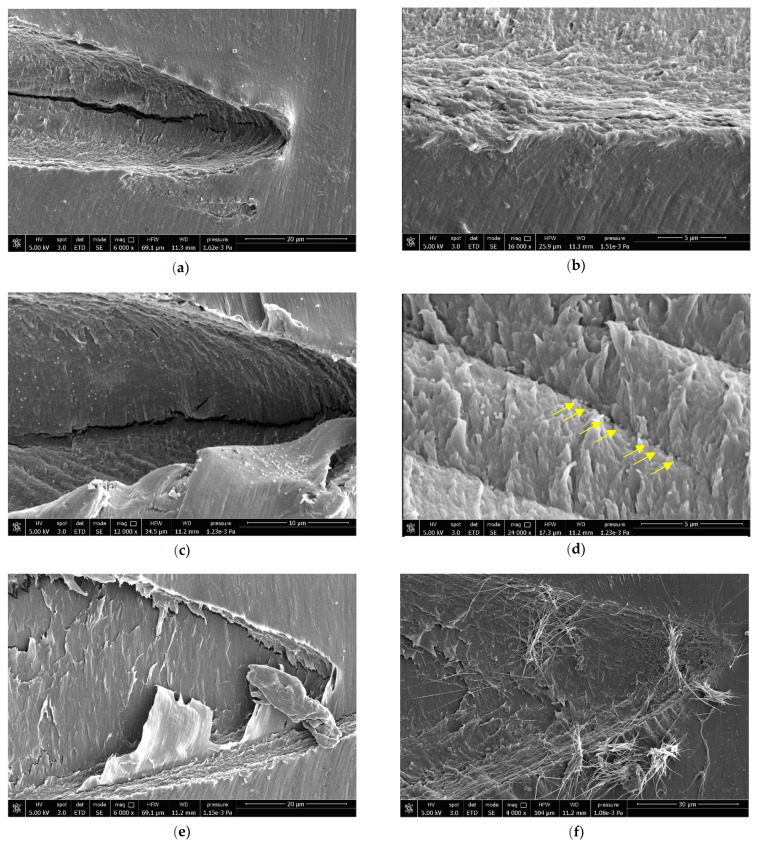
SEM micrographs of the arrested crack surface of the SEDN-4PB: (**a**,**b**) P10, (**c**,**d**) P20, (**e**) S10, and (**f**) S20.

The SEM micrographs of the surface area of the subcritical cracks for all blends are reported in Figure 5. 

The arrested cracks of the P blends results were well-defined and linearly propagated; no preferential paths for the propagation could be recognized, as shown in Figure 5a,c. The high magnification crack image of the P10 compound (Figure 5b) showed crack propagation due to the formation of bands parallel to the path crack. The micrographs of the P20 blend (Figure 5c,d) results were very interesting: first of all, it was easy to recognize the spherical particles of the dispersed PBAT, which were present in greater quantities than in the P10 compound. In addition, the deformation and crack propagation bands were more easily visible and clearly defined (Figure 5c). In particular, in Figure 5d, it can be observed that the PBAT dispersed particles were arranged in rows (as marked by yellow arrows) that follow the course and shape of the band. Moreover, PBAT particles were observed at the apexes of the PLA matrix fibrils. On the basis of what has been observed, it is possible to assume that these deformation bands were associated with the detachment of PBAT particles, having low adhesion with the PLA matrix (as observed in the morphological analysis in Section 3.1).

The arrested cracks of the S blends (Figure 5e,f) showed a different morphology from the P blends (according to the morphological analyses shown in Section 3.1). For S10, it is possible to distinguish a “layered” structure that is not as well-defined for the arrested crack of S20. Furthermore, the S20 micrograph shows several crack propagation lines in a large area in the front of the crack tip, indicating a greater ability of the POE-g-GMA to dissipate energy during the bending test. In Figure 5e, the fibrous nature of the rubbery POE-g-GMA domains can be observed for the S10 mixture (in accordance to the morphological results); in particular, near the crack tip, it can be clearly observed that these rubbery domains are stretched and hooked to the upper and lower sides of the advanced fracture. The mixed POE-g-GMA morphology, consisting of fibrous and spherical domains, can also be noted in the S20 micrograph (Figure 5f) surface. 

At the tip of the arrested crack, both fibres and spheres are present; it is clear that the fibrous domains have been stretched during the mechanical deformation process similarly to the S10 blend. During the deformation, the stretching of the matrix and of the rubber domains occurred. 

#### 3.2.2. TOM Results

The crack propagation is commensurate with the formation and growth of a process zone surrounding the crack [57,58,59,60,61]. The process zone in front of a crack is considered to be a zone of allotropically transformed material: the deformation process thins the sample in the area of the crack tip and the thinning zone at different stages can be observed in Figure 4. The characterization by polarized light microscopy was carried out for all blends. The birefringent zones can be observed under cross polarizers. The TOM micrographs appear brighter and are generally associated with interference colours due to the optical path of the polarized light that is lengthened or shortened. 

**Figure 6:**
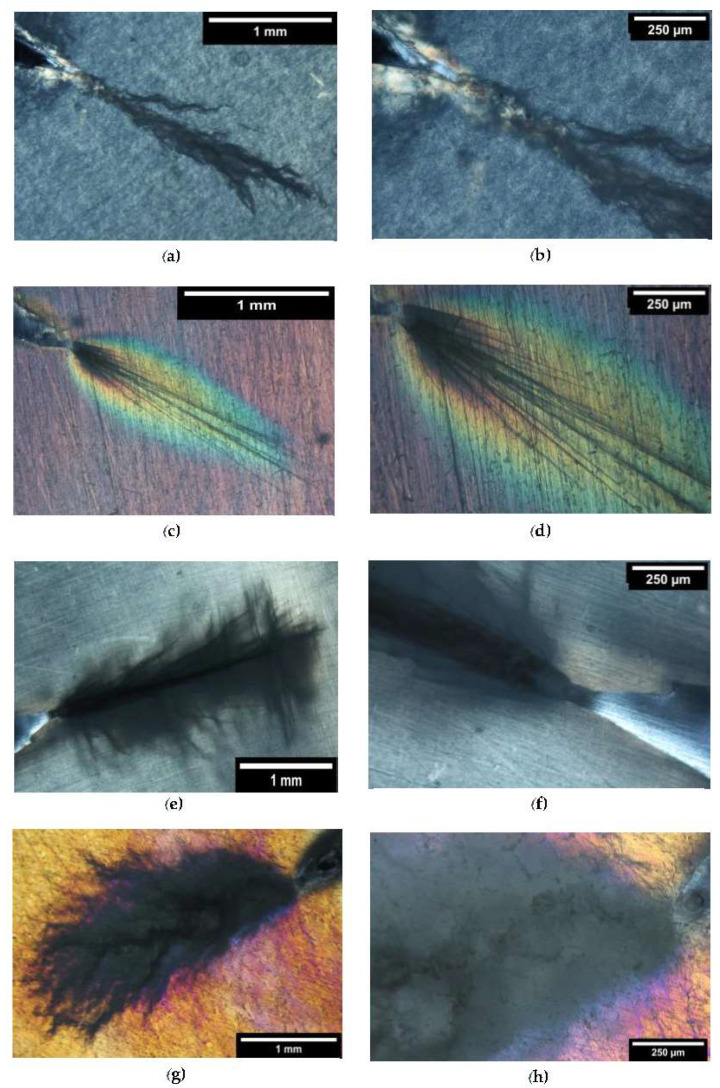
Cross-polarized TOM micrographs of the subcritical crack tip section after deformation for (**a**,**b**) P10, (**c**,**d**) P20, (**e**,**f**) S10, and (**g**,**h**) S20.

Although, for both compounds with 10 wt.% of the rubber, the plume is visible; from the micrographs shown in Figure 6a,b,e,f, it is evident that the appearance of the crack propagation was different for the two compounds. The plume reported in Figure 6a propagates linearly, with little jaggedness and a minor extension of the plastic zone. In contrast, the plume reported in Figure 6e shows a greater extension of the plastic zone and great jaggedness. For both P10 and S10, the process zone could be observed, especially at the tip of the crack. At higher magnifications, a change of colour and brightness, even within the plume of the deformation zone, could be detected. The crack propagation area was more defined for the P10 blend rather than for the S10 blend and it was more difficult to recognize the branches visible in P10. The plume shape change can be correlated to the difference of the dispersed phase: spheres for PBAT and a mix of fibres and spheres for POE-g-GMA. 

The most interesting results, however, were obtained with blends containing 20 wt.% of rubber. The appearance of a deformation before the crack tip changed drastically for the P20 blend: the plume observed in P10 had disappeared, leaving its place and moving to clearly visible and defined branches (Figure 6c,d). The thinned process zone, observed for the first time by Chudnovsky et al. [61], was then clearly observable by the noticeable colour change, with a great deal of birefringence in the first zone in front of the tip. The plastically deformed zone had a large area, showing how much the toughening process, due to the dispersed PBAT phase, was able to disperse the energy released during the deformation phase.

Ragarding the S20 blend (Figure 6g,h), it could be observed that the plume in the crack propagation zone retained a similar shape to the S10 compound but increased in size. The presence of birefringence around the plume can be noticed; a halo of different colours (blue/purple) was observed: the plastic deformation zone affected the whole subcritical crack. 

#### 3.2.3. TEM Results

The TEM investigation was carried out only on the blends containing 20 wt.% of rubber for which, on the basis of what was observed through the aforementioned characterizations, the rubber-toughening mechanisms were more evident and therefore more clearly detectable by TEM. 

**Figure 7:**
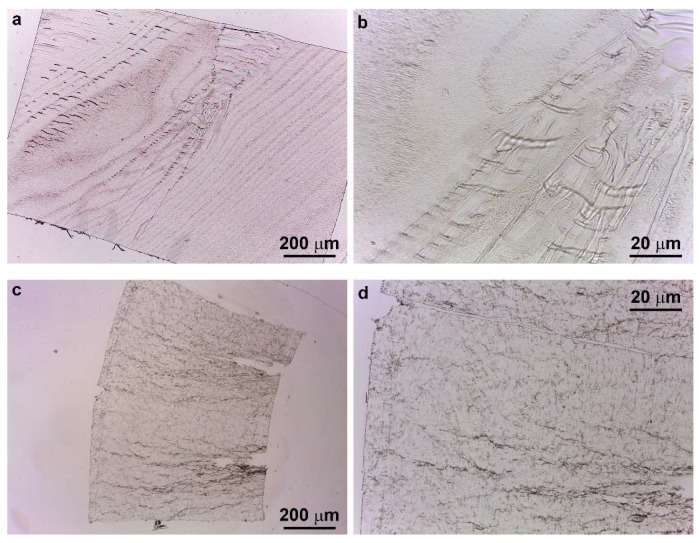
TOM images of the subcritical crack tip section after deformation for the (**a**,**b**) P20 and (**c**,**d**) S20 samples.

In Figure 7a–d, TOM sections of the crack tips, after the SEDN-4PB tests, are shown for P20 and S20 samples, respectively. It is possible to recognize the same branch typology and ramifications observed with the polarized light microscope. 

**Figure 8:**
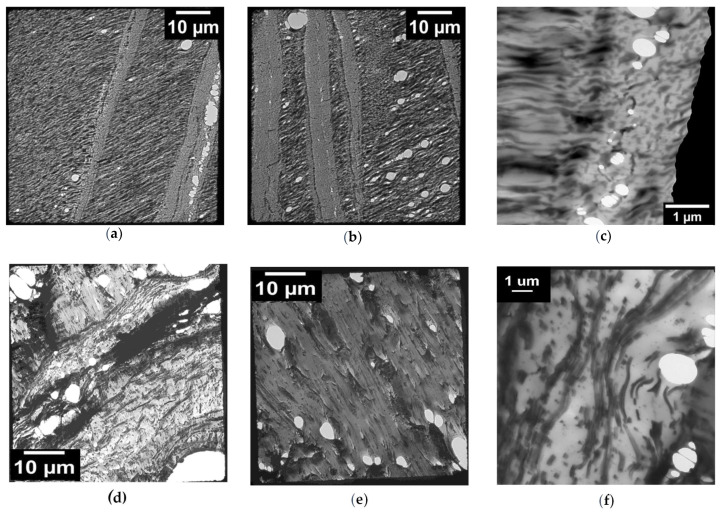
TEM micrographs of the subcritical crack tip section after deformation for the (**a**–**c**) P20 and (**d**–**f**) S20 blend.

The TEM analysis of the P20 blend revealed the presence of cavitated rubber particles located around the crack tip and arranged in a row, confirming the presence of dilatational bands (Figure 8a–c). However, it could be observed that the predominant deformation mechanism at the crack tip seems to be mainly due to shear yielding. The results observed are in line with what was observed in literature for other systems [14,32,62]. On the basis of these observations, for the P20 blend, the main toughening mechanism was the cavitation/debonding of rubber particles, followed by the void growth and induced shear yielding of the matrix caused by the overlapping of dilatational bands [19,63]. This sequence of micromechanical deformation mechanisms well explains the macromechanical results obtained where a high increase in the elongation at break (slow test ductility) was registered.

A similar but not equal behaviour was observed for S20 (Figure 8d–f). As the mixed fibrous/particle morphology of S blended, the cavitation/debonding of the rubber particles was less extended and zones of plastic deformation of the fibrillary parts were observed. The deformation of these zones is most likely responsible for the different deformations at the crack apex also registered in the TOM images. For this blend, the micromechanical deformation mechanism associated with the toughening is therefore mainly related to the plastic deformation of the fibrous nature of POE-g-GMA. The micromechanical deformation mechanisms associated with the S blends’ morphology were mirrored macroscopically into a greater impact resistance. 

The results of the TEM magnifications, in which the simultaneous presence of cavitation and debonding phenomena were highlighted, can also be confirmed by the application of analytical models correlated to the yield behaviour of the blends. Indeed, for binary blends that present fully cavitated particles, Lazzeri et al. [15] demonstrated that yield stress follows Equation (1):

(1) $$\sigma_{y}=\left(1-1.375\varphi_{dispersed\;phase}\right)\sigma_{0}\;\;$$

where *σ_y_* and *σ*_0_ represent the experimental stresses at the yield of the binary blend and matrix, respectively, and *φ* is the volumetric fraction of the dispersed phase.

In addition, for the binary blends that present fully debonded particles, the stystes behave as a particulate rigid-filled material following the Ishai-Cohen equation [64] (Equation (2)):

(2) $$\sigma_{y}=\left(1-1.21\varphi_{dispersed\;phase}{}^{2/3}\right)\sigma_{0}\;\;$$

**Figure 9:**
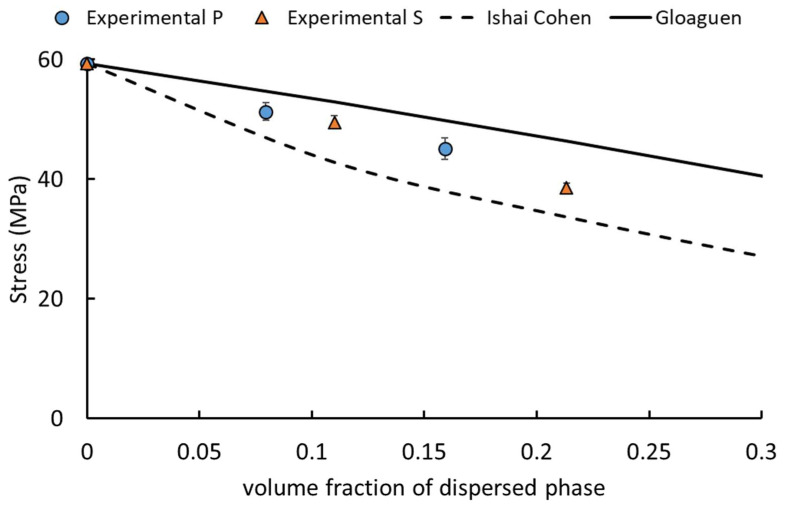
Stress at the yield variation with increasing of the volume percentage of PBAT: experimental data compared with predictive analytical models.

As shown in Figure 9, the experimental data lie among the upper and lower bounds constituted by Equations (1) and (2), demonstrating that not only a single mechanism was present during the tensile stress application but the rubber particles underwent cavitation and debonding concurrently.

## 4. Conclusions

In this work, the rubber-toughening mechanism occurring in two different injection-molded PLA-elastomer blends were investigated. In total, 10 and 20 wt.% of PBAT and POE-g-GMA were introduced into a PLA matrix.

From a mechanical point of view, PBAT increases mainly the elongation at break while POE-g-GMA increments the Charpy impact resistance. Through SEM investigations, the nature of the dispersed phases emerged: spherical well-dispersed PBAT particles and a mix of spheres/fibrils for POE-g-GMA were present. 

To better understand the toughening mechanisms, the coupling of the SEDN-4PB technique and different microscopical analyses (SEM, polarized TOM, and TEM) were carried out, for the first time, on PLA-based blends. 

Analyses under polarized light microscopy showed, for both types of PLA blends, a large area of plastic deformation in the front of the crack tip. The plastic zone, which is birefringent, increases as the percentage of dispersed rubber increases.

TEM observations carried out on blends with 20 wt.% of elastomer revealed the main differences among the P and S blends. Even if, for both formulations, the damage is mainly generated by shear yielding deformation, although voids associated with dilatational bands are observed, the different rubber morphology influences the final micromechanical deformation mechanisms. For PBAT-based blends, as they are spherical, the cavitation/debonding of rubber particles, followed by void growth and the induced shear yielding of the matrix caused by the overlapping of dilatational bands, are the main toughening sequences that cause a high increase of elongation at break.

In contrast, for POE-g-GMA, being a spherical/fibrillar mix, the micromechanical deformation mechanism associated with the toughening is therefore mainly related to the plastic deformation of its fibrous nature, macroscopically generating a high impact resistance. 

## Figures and Tables

**Table 1:** Blend names and compositions.

Blend Name	PLA (wt.%)	PBAT (wt.%)	POE-g-GMA (wt.%)
P10	90	10	0
P20	80	20	0
S10	90	0	10
S20	80	0	20

**Table 2:** The injection-molding processing parameters adopted for all blend compositions.

Parameter	Value
Temperature profile from feeder to the injection zone (°C)	180/180/185
Mold temperature (°C)	60
Injection holding time (s)	5
Cooling time (s)	25
Injection pressure (bar)	90

**Table 3:** Mechanical properties of the PLA/PBAT and PLA/POE-g-GMA blends.

Formulation	Elastic Modulus (GPa)	Stress at Yield (MPa)	Stress at Break (MPa)	Elongation at Break (%)	Tensile Toughness (MJ/m^3^)	Charpy Impact Strength (kJ/m^2^)
PLA [56]	3.4 ± 0.2	/	59.4 ± 1.2	3.8 ± 1.5	1.05 ± 0.1	3.0 ± 0.4
P10	2.8 ± 0.1	51.3 ± 0.5	26.2 ± 0.6	9.3 ± 2.5	3.10 ± 0.3	3.9 ± 0.8
P20	2.0 ± 0.1	45.1 ± 1.8	28.1 ± 1.5	276.3 ± 8.1	62.76 ± 5.2	4.7 ± 0.2
S10	2.8 ± 0.1	49.5 ± 1.0	37.5 ± 3.1	4.5 ± 0.2	1.14 ± 0.1	5.6 ± 0.6
S20	2.3 ± 0.1	38.6 ± 0.5	30.1 ± 2.3	20.9 ± 4.6	5.84 ± 1.3	9.4 ± 1.5

## Data Availability

Data presented in this study are available upon request from the corresponding author.
